# The Radcliffe Infirmary, Oxford

**Published:** 1894-11-24

**Authors:** 


### The Institutional Workshop.

### WITHIN THE HOSPITALS.

### THE RADCLIFFE INFIRMARY, OXFORD.

By Our Special Commissioner.

In The Hospital for April 11th, 1891, and in subsequent issues this institution was brought to the test of criticism, and found wanting in many particulars. Since then several thousand pounds have been collected, and the committee have set themselves to work to make certain structural additions and alterations, and to generally put their house in order. Having received an invitation to visit the hospital again, we had the pleasure of doing so on the 1st inst., and were met by the Treasurer (Lord Dillon), the Rev. C. J. H. Fletcher, and the Matron, and under their escort we proceeded to visit the whole institution. It will be remembered that our criticisms in 1891 were grouped under eight main heads, and, with the view of testing to what extent the necessary reforms have been carried out, we propose to give them seriatim. Our criticisms then were directed: (1) against the condition of the walls, baths, and other appliances, and especially the state of the Parian cement in the chief surgical ward; (2) the unworthy appearance of the staircase and the entrance hall of the Jubilee Wing, owing to their not having been painted; (3) the general absence of regard for appearances in many portions of the institution; and (4) certain inadequate fittings which it contained. Our visit showed that the money spent by the committee, and the changes they have introduced, have remedied, and, in the main, removed the causes of criticism under the above four heads, with the exception of certain fittings. The main surgical ward, for instance, is now a handsome ward, clean, bright, and pleasant to the eye; indeed, the improvement was so marked as to make it difficult to recognise it again. The whole institution has been put into thorough order internally, so far as those portions are concerned which are occupied by the patients. The result is, on the whole, satisfactory, and the changes effected are so considerable as to make the hospital proper much more worthy of the University and city of Oxford than it has probably ever been in its whole history. Our visit has convinced us that there has been a considerable awakening, and there was evidence of an earnest desire on the part of those responsible to promote improvement and increase the efficiency of the hospital. We have, therefore, much pleasure in congratulating the late treasurer, Dr. Bright, and the present treasurer, Lord Dillon, and their colleagues upon the excellent work they have done in the directions indicated, and we take this opportunity of thanking the Treasurer, Mr. Fletcher, and the Matron for their courtesy on the occasion of our visit. We take the improvements everywhere noticeable at the Radcliffe Infirmary as proofs that the committee will not rest satisfied with the present state of the institution, but that they will go forward with courage and determination until the whole of the necessary changes have been made, not only in the buildings, but in the administration also.

Further Building Operations Necessary.

There were four other matters which we criticised in 1891, which still remain practically in the same state as when we paid our first visit in that year. These were: (5) The condition of the basement and other parts of the oldest portion of the buildings; (6) the general aspect presented to the visitor on entering; (7) the fact that the accommodation provided for the nurses is altogether behind the times, especial as regards the cubicles and bathing arrangements; (8) the lack of active public interest in the infirmary. We spent a good deal of time in, and have given much consideration to the condition of the main block, *i.e.*, the oldest building which faces the Woodstock Road. This block was condemned by Howard a century ago, and, after careful examination of the vacant wards, their floors, and walls, and the accommodation provided for the nurses; after realising that this old, out-of-date and unattractive building blocks out light and air, and prevents the people of Oxford from seeing the better and more attractive portions of the Infirmary buildings, we have come to the conclusion that it must be pulled down entirely, because such a course is not only the best from a hygienic and artistic point of view, but also because it will ensure a much wider popularity to the hospital, and enable a new and attractive administrative wing to be erected of modern design, replete with every possible improvement, at a cost little if at all greater than the sum which would be required to gut the old building and reconstruct it. It would be unfair, if not dangerous, to put the nurses and other members of the staff to reside within the portions of this building formerly occupied by patients, unless or until the internal walls had been stripped and removed, and the whole of the floors, fittings, woodwork, fireplaces, *i.e.*, practically everything, taken out and carted away. Such a reconstruction as this is not only costly, but it would entail risks in regard to structure which might result in necessitating the pulling down of the outer walls also. Besides, the annual outlay on such a building and the expenses of its administration would be infinitely greater than in the case of a modern structure, whilst the accommodation could never be made entirely satisfactory or up to date. For these reasons we cannot doubt that the committee will be wise to condemn this building, to have plans prepared for a new administration block, and to set about the work without any further delay. It is certainly matter for serious anxiety that the whole of the nursing staff should be so badly housed at present as to render it well-nigh impossible to hope that they can long continue to occupy their present quarters without serious risk to health. That this is not too strong a statement will be admitted when it is pointed out that 21 nurses occupy cubicles and rooms at the top of the old building, where there is only one bath for the use of the whole of them, and where the cubicles communicate directly with the lavatories and w.c.'s, so that the atmosphere they breathe must necessarily be anything but pure or desirable, especially when the state of the weather necessitates the closing of ventilators and windows in their sleeping apartments. It must further be recognised that the rooms occupied by the sisters in the newer buildings attached to the wards are not desirable, though they may be tolerable, as sleeping rooms for women who spend their lives in sick wards, where many of the cases are severe. The outlook from these sisters' rooms is unattractive; the windows have a nothern aspect, and, apart from these drawbacks, it cannot be doubted that it is desirable for each sister to sleep away from her ward, so that she may get necessary change and entire rest when off duty. These considerations lead us to urge the committee to hesitate no longer, but to resolve forthwith to prepare plans for a new administration block containing adequate accommodation for the nursing and resident staff.

### The Financial Aspect.

We can quite understand that the first impression which the foregoing suggestions will make upon the mind of the average economist in Oxford will be that it is impossible for the committee to face an expenditure of from £5,000 to £7,000, which is about the sum which would probably be required to do the work of reconstruction here recommended. It must be remembered, however, that £5,000 was the sum which Dr. Bright assigned to this work at the Quarterly Court of Governors on April 29th, 1891. We are not aware how much money the committee have in hand, being the balance of the sum raised in consequence of the appeals issued during recent years. We find, however, from the report, page 48, that the Radcliffe Infirmary has an invested capital of £64,000, and that the ordinary expenses for the year 1893 were under £7,500. It is perfectly certain that if the Radcliffe Infirmary had had less invested funds it would have had a much larger income to-day than it has at present. Experience shows that it is a danger, rather than a safeguard, to an old charity to have an invested capital of a greater amount than a sum represented by, say, five years' expenditure. An invested capital of £40,000 is therefore, in our judgment, the maximum sum which it is necessary for the Radcliffe Infirmary to have to make it perfectly secure financially, and to enable it to discharge its duty to the community with the utmost efficiency. We do therefore most earnestly urge the committee to move the Governors to sanction a sale of sufficient capital to enable them to proceed forthwith to build the new administration block, and so to bring their institution up to the highest modern standard of hospital efficiency. Failing this, having a reserve of so large an invested capital as £64,000 behind them, in addition to land and buildings, we would suggest that they should follow the example of some other hospitals and nurse-training schools by issuing 4 per cent. debenture bonds to the extent necessary to yield the capital sum required, to cover the entire cost of the new administration building. The interest upon these bonds and a sinking fund should be made a first charge upon the payments received from the nursing department, which, with the new buildings, could be put upon a basis which would make the income derived from this source considerably larger than it is at present. During the year ending December 31st, 1893, the hospital received £361 in payments from its probationers and private nurses. This sum would suffice to provide 4 per cent. interest and a sufficient sinking fund for the debentures. If so large a sum can be earned by the hospital from its training school under present circumstances, the income which would come in, were the committee to provide the nurses with good accommodation, a three years' course, well-organised lectures and instruction, and a first-class certificate, would certainly amount to at least £1,000 per annum. We were glad to gather from our conversation with some of those who take an active interest in the progressive development of the Radcliffe Infirmary that the views we here venture to express are in accordance with those they themselves hold. Once remove the old administration block and erect in its place a modern building which, like the Nurses' Home at the Royal Infirmary, Edinburgh, should be a model for the imitation of other hospitals throughout the world, and the public attention which will be directed to this institution will be so great as to secure a revenue large enough to enable the committee to put money by every year, instead of, as at present, leaving them with a balance on the wrong side to carry forward into the next year's accounts. We insist so strongly upon this point because we are confident that, when this radical and pressing alteration has been made, it will be no longer possible for the experienced visitor to note with regret the absence of much real interest in this institution on the part of the inhabitants of Oxford.

### Changes in the Administration Recommended.

We have spent so much time and thought upon this institution that its progressive development and well-being have become vitally interesting to us, and we desire, therefore, to do our best to make it what it ought to be—the admiration of all who take an intelligent interest in the best administered hospitals of the world. During our visit we came to the conclusion, as we did in 1891, that the committee would be well advised to appoint a special committee to go into the bye-laws, rules, and orders, with a view to revising the general administration of the institution. At the present time the hospital sadly needs an active permanent trained head of its executive staff. We are strongly of opinion that the committee would be well advised to place the whole of the internal administration in the hands of a medical superintendent or principal medical officer, and to give him the assistance of a house physician and house surgeon. At the present time, under the existing conditions, there is within the building an absence of medical authority of sufficient influence, force and continuity to ensure the maximum of efficiency in various directions. As a consequence, we are inclined to believe that certain matters exist in the children's ward which affect the well-being of the patients, which ought to be remedied, and which would be remedied by such a change of system. No committee which fails to secure and follow the advice of a capable medical administrator in the internal management of the hospital under its administration is in reality fit to have charge of such an institution. Many excellent laymen, who are good administrators, fail from ignorance to recognise the evils attaching to the absence of a recognition of this important principle on the part of a hospital committee. We would therefore very earnestly recommend the adoption of the following changes in the system at Oxford. First, to place the whole internal administration under the responsible control of a principal medical officer or superintendent, who would co-operate with the matron in organising the nurse-training school, and, at the same time, attend to the hygiene and comfort of the resident staff as well as of the patients. He should be assisted by a qualified house surgeon and house physician, who might be appointed for twelve months, and receive an honorarium in addition to their board and residence. The work in the out-patient department and the unusually large number of books which the two resident medical officers have to keep at present make such a change desirable from the aspect of the work to be done alone, while, in the interests of good administration, and on many other grounds, the sooner such a change is made the better. Next, in our view it is desirable that there should be a secretary who has had a considerable training in hospital management, and who has sufficient capacity to finance the institution; that is to say, to increase its income at once, say, to £10,000 a year, so that the committee may have ample funds to keep it in perfect order and make it a model—as the hospital of Oxford University ought to be a model—of everything that is best and, from the highest standpoint of science, necessary too. The absence of such an officer cripples the hands of a committee in many ways, and we are confident that the increased cost of this change in the present system would be so small as to be relatively inappreciable, especially having regard to the larger funds which would then certainly be available for the purposes of the hospital. We are further of opinion that the matron should have the assistance of a capable housekeeper, who should be responsible for the proper quality of the food, its preparation for the table, and the general catering for the whole establishment. Where the matron, as at present, has all this work to do in addition to looking after the nursing and domestic staff, as well as the supervision of the whole institution, it is impossible that things should always go satisfactorily. Hence, rightly or wrongly, feelings of dissatisfaction and possibly of discomfort may arise from time to time in the minds not only of the resident staff, but of the patients and their friends. For all these reasons, and for others which we have not space to state on the present occasion, we do most earnestly urge the committee to at once appoint a special committee, and to take competent advice, so that they may consider carefully this question of a reorganised administration, which we are inclined to think is only a little less important, if it is even of less importance than the pressing necessity which undoubtedly exists for the removal of the old administration block, and the erection upon its site of the new buildings which we have fully referred to above. If it were possible to remove the old front building and to erect a new administration block by some instantaneous process everybody would marvel at the immensity of the improvement, and all would be proud of the Radcliffe Infirmary, because it would then be a model institution worthy of the highest and best traditions of our oldest and greatest University, its city, and county.

